# Advanced Electron Microscopy of Nanophased Synthetic Polymers and Soft Complexes for Energy and Medicine Applications

**DOI:** 10.3390/nano11092405

**Published:** 2021-09-15

**Authors:** Jihua Chen

**Affiliations:** Center for Nanophase Materials Sciences, Oak Ridge National Laboratory, Oak Ridge, TN 37831, USA; chenj1@ornl.gov

**Keywords:** nanostructures, polymers, organic crystals, electron microscopy, energy, medicine, AI (artificial intelligence)

## Abstract

After decades of developments, electron microscopy has become a powerful and irreplaceable tool in understanding the ionic, electrical, mechanical, chemical, and other functional performances of next-generation polymers and soft complexes. The recent progress in electron microscopy of nanostructured polymers and soft assemblies is important for applications in many different fields, including, but not limited to, mesoporous and nanoporous materials, absorbents, membranes, solid electrolytes, battery electrodes, ion- and electron-transporting materials, organic semiconductors, soft robotics, optoelectronic devices, biomass, soft magnetic materials, and pharmaceutical drug design. For synthetic polymers and soft complexes, there are four main characteristics that differentiate them from their inorganic or biomacromolecular counterparts in electron microscopy studies: (1) lower contrast, (2) abundance of light elements, (3) polydispersity or nanomorphological variations, and (4) large changes induced by electron beams. Since 2011, the Center for Nanophase Materials Sciences (CNMS) at Oak Ridge National Laboratory has been working with numerous facility users on nanostructured polymer composites, block copolymers, polymer brushes, conjugated molecules, organic–inorganic hybrid nanomaterials, organic–inorganic interfaces, organic crystals, and other soft complexes. This review crystalizes some of the essential challenges, successes, failures, and techniques during the process in the past ten years. It also presents some outlooks and future expectations on the basis of these works at the intersection of electron microscopy, soft matter, and artificial intelligence. Machine learning is expected to automate and facilitate image processing and information extraction of polymer and soft hybrid nanostructures in aspects such as dose-controlled imaging and structure analysis.

## 1. Introduction

Electron microscopy has been of continued interest for decades, playing vital roles in revealing micro- and nanostructures as well as functionality relationships in traditional and novel materials. Synthetic polymers, traditionally including four major industrial categories (adhesives, elastomers, coatings, and fibers), have intriguing structures that are often hierarchical in nature and span from the nano- to micrometer scales [1]. Synthetic polymers and their small molecule counterparts such as organic crystals and amorphous or crystalline oligomers, as well as their interfaces and composite complexes with inorganic materials, now find their impacts well around us in applications such as energy storage, energy harvesting, ion and electron transport, sensors, and medicines [1,2,3,4,5]. Compared to inorganic materials, synthetic polymers and soft complexes yield poor contrast in electron microscopy due to their intrinsically lighter elements (C, N, S, O, F, H, etc.) and severe electron beam-produced changes. In addition, it is well known that electron microscopy of synthetic polymers has very different expectations and challenges (for example, morphological variations) than that of proteins, DNAs, and other biomacromolecules with monodispersity and consistent 3D structures [6]. Two older [7,8] and two newer reviews [6,9] summarized the progress in electron microscopy of synthetic polymers and organic materials over the years.

Since 2011, the Center for Nanophase Materials Sciences (CNMS) at Oak Ridge National Laboratory has been working with numerous facility users on nanostructured polymer composites, block copolymers, polymer brushes, conjugated molecules, organic–inorganic hybrid nanomaterials, organic–inorganic interfaces, organic crystals, and other soft complexes. This review crystalizes some of the essential challenges, successes, failures, and techniques during the process in the past ten years. It also presents some outlooks and future expectations on the basis of these works. Machine learning is expected to automate and facilitate image processing and information extraction of polymer and soft hybrid nanostructures in aspects such as dose-controlled imaging and soft structure analysis.

## 2. Comparison of Various Nanostructure Characterization Techniques

**Figure 1:**
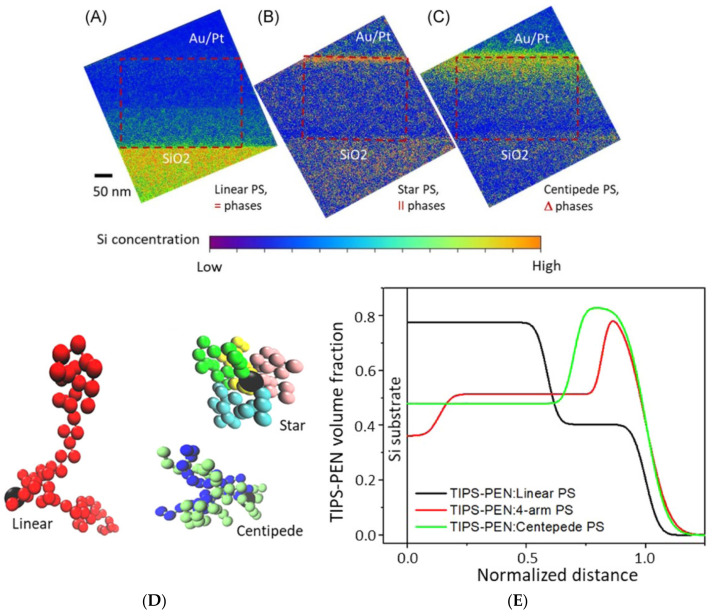
Cross-section EFTEM Si element mapping reveals through-thickness concentration profiles of Si-containing TIPS-PEN in blend films with various PS binders: (**A**) linear, (**B**) 4-arm star, and (**C**) centipede. The interface of each blend film with SiO_2_/Si is referred to as the bottom, while the top surface (also referred to as an air interface) is coated with Au/Pt. Images (**A**) and (**C**) clearly visualize vertical (=) and gradient (Δ) phase segregation, respectively, while mapping of a TIPS-PEN-rich domain in a lateral (II) star PS blend film (**B**) shows a high TIPS-PEN concentration at the top. EFTEM, energy-filtered transmission electron microscopy; PS, polystyrene; TIPS-PEN, 6,13-bis(triisopropylsilylethynyl)-pentacene. (**D**) Typical bead conformations of three PSs. The central beads are colored black in all three, while side chain beads are represented by different colors in the star and centipede models. (**E**) Volume fraction profiles of TIPS-PEN in blend films, calculated from neutron reflectivity. Distance from the substrate surface is normalized against film thickness. TIPS-PEN, 6,13-bis(triisopropylsilylethynyl)-pentacene. Reproduced from [10] under the terms of the Creative Commons CC BY license [18]. No changes have been made.

The current generation of novel polymers and soft complexes often exhibits hierarchical structures that are worth exploring from the millimeter to nanometer scales, which account for their cutting-edge applications in energy, medicine, and numerous other fields [3,6,7,8,9,10,11,12,13,14,15,16,17]. For example, complicated structures of nanophased polymer and soft complexes for biomimetics [16] and optoelectronics [17] easily span over length scales of five or six orders of magnitude. This marks the importance of comprehensively understanding their structure–property relations with a host of length scales and dimensionality. Figure 1 is an example of using a combination of X-ray, neutron, scanning probe, and electron microscopy in studying the crystallization of a benchmark small molecule organic semiconductor, 6,13-Bis(triisopropylsilylethynyl)pentacene (TIPS-PEN), in the presence of a polystyrene additive with varied chain architectures (star, centipede, and linear) for charge transport improvements [10]. In Figure 1A–C, cross-sectional silicon mapping reveals vertical phase separation of TIPS-PEN/polystyrene composite layers on silicon wafer substrates that have thermally grown SiO_2_ layers.

### 2.1. Probes: Tip, Electron, Ion, X-ray, and Neutron

The probes that we use can play a fundamental role in the nanostructural information extraction process. A previous review on nanostructures of organic solar cells elaborated on the differences of various common probes in terms of their strengths and limitations [2]. Scanning probes are based on tip–sample interactions and rely on close physical proximity, thus belonging to surface characterization techniques, which can be distinguishable from the other probes that are discussed here. Electrons, ions, X-ray photons, and neutrons are of variable energies and penetrating powers. Among these four penetrating probes, the interaction intensities between the probe and sample generally decrease in order from ion, through electron and X-ray, to neutron [2]. In other words, electron microscopy can induce significant sample changes more quickly than X-ray or neutron experiments. At the same time, if the beam-induced changes can be monitored and managed effectively, electron microscopy may offer nanostructural information more speedily.

Electron–polymer interaction is acceleration voltage-dependent [8], and the resistance of polymers to electron beam-produced microstructural changes is proportional to their thermal stability, which can be a function of their melting temperature, glass transition temperature, or degradation temperature [7,8]. For example, synthetic polymers with π–π structures are more likely to maintain their structural integrity under the same electron beam exposure than those with only C–H_2_ backbones [7]. How to deal with the higher sample–probe interaction remains the key to success in nanostructural analysis of synthetic polymers and soft complexes in electron microscopy.

The probe size generally increases in order from electron, through X-ray, to neutron [2]. This means the sampling area (or statistically averaged area) increases from electron, through X-ray, to neutron. The highest achievable spatial resolution may not be the most important factor to consider in many cases because there are many other factors that come into play, such as sample preparation with proper structural preservation, the length scale transition in hierarchical structures, the match of features and the sampling area, and the differences between the surface and bulk morphology due to nanoconfinement effects.

According to the acceleration voltage-dependent wavelength of an electron,

(1) $$\lambda =\frac{h}{\sqrt{2meV}}$$

(h is Plank’s constant, m is the electron mass, e is the electronic charge, and *V* stands for the acceleration voltage of the microscope), the wavelength of electrons in electron microscopes is 0.017 nm for 5 kV, and 0.0027 nm for 200 kV, as compared to the infrared wavelength of 700–1000 nm, the visible light wavelength of 400–700 nm, the ultraviolet light wavelength of 100–400 nm, and the copper K-alpha wavelength used in X-ray diffraction of 0.154 nm, as well as a typical neutron wavelength in neutron scattering or diffraction experiments on the order of 0.1 nm [2,7,8].

### 2.2. Connecting Real and Reciprocal Space

Nanostructural characterization techniques in real space are highly complementary to those in reciprocal space. There is a necessity to confirm the nanostructural information extracted from one and the other.

Scanning probes are intrinsically real-space techniques, although efforts are underway to combine other characterization tools with them. X-ray and neutron are intrinsically reciprocal space techniques but can be converted into real-space microscopy-type variations. Electron microscopy covers both real and reciprocal space techniques [2,6,7,8,9,10,19] by itself, offering a wide range of experiments at sample surfaces (scanning electron microscopy, SEM, and energy-dispersive X-ray spectroscopy, EDX), or the cross-sample thickness (transmission electron microscopy, TEM, and scanning transmission electron microscopy, STEM). Energy loss features can be used for electron energy loss spectroscopy (EELS). With pixel-by-pixel signal analysis, it is possible to use energy-filtered TEM (EFTEM) to map the features of electron energy loss spectra [6,9,20]. Selected area electron diffraction (SAED) and dark-field (DF) imaging may be used in parallel to achieve nanoscale characterization in both reciprocal and real space, if the experiments can be performed before significant structural changes happen under the electron beam.

### 2.3. Cryotome, Cryo-Holder, Cryo-Chamber, and Low Dose

For electron microscopy of synthetic polymers and soft complexes, the prefix “cryo” appears frequently, which requires additional attention and clarification.

A cryotome is a microtome tool for samples with glass transition temperatures lower than room temperature [6,7,8,9,20]. For example, elastomers, gels, rubbery materials, and cells may require a cryotome to preserve their natural structures and prevent deformation during the sample preparation process.

A cryo-holder is useful in lowering the sample temperature during TEM experiments. By a cryo-holder alone, the sample temperature during TEM experiments may not be able to reach and maintain the liquid nitrogen temperature [6,7,8,9,20]. A surrounding cryo-chamber inside the TEM itself is usually designed for carrying out this trick, which is a requirement for cryo-plunged or fast-frozen liquid-form samples.

The cryo-temperature can slightly slow down the lattice parameter expansion and degradation of beam-sensitive materials under electron beams; however, it may not extend the end-point dosage significantly [19]. Thus, the importance of low-dose imaging may not be ignored, as it aims at acquiring the maximum amount of nanostructural information with the lowest amount of electron dosage delivered to the sample area. As the magnification increases, the electron dose delivered will increase by the power of two [8]. Identifying the changing nanostructures under an electron beam, monitoring them closely, and damage mitigation are always at the center of the challenging process of electron microscopy for synthetic polymers and similar materials.

## 3. Sample Preparation Based on Contrast Mechanism

The contrast mechanism in synthetic polymers and soft complexes plays a vital role in planning their microscopy experiments from the very beginning. Choosing a different contrast mechanism may mean a different type of TEM grid and grid coating, another thought on the stain, and other changes in sample preparation.

For synthetic polymeric and soft complexes, there are four main characteristics that differentiate them from their inorganic or biomacromolecular counterparts [6,7,8,9]:

(1) Lower contrast as compared to inorganics;
(2) Abundance of light elements as compared to inorganics;
(3) Polydispersity or nanomorphological variations as compared to proteins;
(4) Large changes induced by electron beams as compared to inorganics.

Items (2)–(4) also contribute to item (1). Item (2) renders EDX difficult for synthetic polymers and soft complexes. Item (3) means techniques for biological cryo-TEM may not be transferable, and sometimes even irrelevant. Item (4) causes every aspect of electron microscopy to be much more challenging, spanning from EELS, EFTEM, SAED, and high-resolution TEM (HRTEM).

Contrast in electron microscopy of synthetic polymers and soft complexes may be satisfactorily generated by the following techniques, although each sample may be a different case [6,8,11,13,14,20,21,22,23,24]:

- Surface- or interface-induced contrast;
- Intrinsic contrast from the increased density of crystalline domains;
- Shadowing with an additional ultrathin coating or epitaxial growth;
- Phase contrast that gives rises to lattice fringes;
- Z contrast by a (high-angle) annular dark-field detector in STEM;
- Inelastic electron scattering (EFTEM contrast) from low-energy plasmon regions and core edges;
- Diffraction contrast using an objective aperture;
- Staining by iodine, a heavy metal, etc.

After deciding on a contrast mechanism, the next step is to generate samples of proper thickness (50–100 nm) and optimal viewing quality without sacrificing real structures.

### 3.1. Focused Ion Beam (FIB) and Microtome

**Figure 2:**
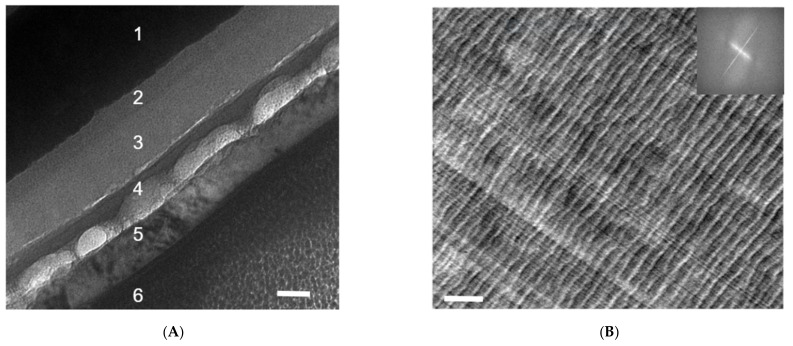
(**A**) A cross-sectional TEM image of an organic solar cell processed by FIB. Layers 1–6 correspond to (1) indium tin oxide (ITO), (2) spin-coated poly(3,4-ethylenedioxythiophene) polystyrene sulfonate (PEDOT:PSS) layer, (3) low-bandgap polymer/PCBM composite layer, (4) calcium layer, (5) aluminum electrode, and (6) platinum protection layer for FIB sample preparation. The black-dotted structures on layers 2 and 3 are likely caused by a polishing current that is to be reduced during FIB sample preparation. The polishing also causes delamination between layers 3 and 4, as well as an uneven thickness of layer 4. The scale bar is 50 nm. This is an unpublished result related to a previously published work [21] (**B**). Wavy microstructures of microtomed PBI films and the cutting lines that are vertical to them. A fast Fourier transformation (FFT) of the left TEM image is presented on the right. The straight thin lines running from top left to bottom right are the results of the non-smooth diamond knife edge, and the wavy curves vertical to them with larger spacings result from the intrinsic mechanical instability of the film. The scale bar is 200 nm.

FIB is the sample preparation method of choice if the interface of interest is buried underneath layers of other materials, or a cross-section view is desired along the direction of the sample thickness (or out of plane) [6,7,8,9]. A FIB operation is often started with deposition of the protective layer and is then followed by fast-paced milling, and gentle final polishing to guarantee that the delicate sample nanomorphology is preserved after ion bombardments and before a lift-off [6,7,8,9,25]. Alternatively, a tilting position may use the substrate as the protective layer in the so-called shadow-FIB method [25]. FIB sample preparation of polymer samples requires a much lower ion beam current as compared to their metal or ceramic counterparts. According to the work by Kim, Liu, and Minor [25], a microtomed polystyrene-*block*-polymethyl methacrylate (PS-*b*-PMMA) slice of 100 nm thickness yielded no significant structural changes with a FIB ion beam current of 100 pA at 30 kV, outside the ion drilling area. However, with an ion beam current of 300 pA or higher, significant distortion, wrinkling, and possible crosslinking can be observed in the polymer thin film due to thermal damage [25]. Figure 2A is a cross-sectional TEM image of an organic solar cell [21] processed by FIB. Layers 1–6 correspond to (1) indium tin oxide (ITO), (2) a spin-coated poly(3,4-ethylenedioxythiophene) polystyrene sulfonate (PEDOT:PSS) layer, (3) a low-bandgap polymer/PCBM composite layer, (4) a calcium layer, (5) an aluminum electrode, and (6) a platinum protection layer for FIB sample preparation. The black-dotted structures on layers 2 and 3 are likely caused by a polishing current that is to be reduced during FIB sample preparation. The polishing also causes delamination between layers 3 and 4, as well as an uneven thickness of layer 4.

A microtome is a common tool for sectioning plastic samples [8,20]. A cryo-microtome is required for materials with a glass transition temperature below room temperature. Microtomed stubs are usually ideal surfaces for atomic force microscopy because a smooth surface may be difficult to achieve for stand-alone soft materials. The microtomed samples are typically floated on filtered water for lift-out with TEM grids [6,7,8,9,20]. A gold color and continuous ribbon formation indicate that slices with a proper sample thickness are likely achieved, although the optimal slice thickness is also dependent on the chemical nature of the sample itself and the purpose of experiments (EFTEM, HRTEM, SAED, etc.) [6,7,8,9,20]. Low-dose HRTEM may require a thinner sample for better beam penetration, while too thin a sample can increase the chance of instability during imaging. A too thick sample may complicate or prevent accurate EFTEM experiments. A slightly thicker sample may improve the intensities of the best acquirable low-dose SAED pattern.

Embedding of thin film or fiber-form samples is often necessary in order to reveal their internal nanostructures as a function of the diagonal or vertical distance from the outer surfaces [8,20]. A low-viscosity, high-strength, two-part epoxy formula is often required to provide a robust matrix around the sample as well as enough adhesion at the epoxy–sample interface. Even with the best possible epoxy embedding, some sample films are intrinsically poor in mechanical performance at 50–100 nm thickness. Tearing, folding, smaller sizes of sliced sections, and an increased electron beam sensitivity should all be closely watched. These may be partly improved by providing a carbon film support underneath the microtomed slices on TEM grids. Drying the TEM grids sideways through a filter paper may prevent fragile slices from breaking apart.

The diamond knife edges of microtome machines require close monitoring and regular maintenance (sharpening) [6,7,8,9,20]. Otherwise, a cracked edge on a diamond knife can induce periodic artifacts or lines on sample slices, which should not be mistaken as the real morphology of the sample. Figure 2B shows a microtomed slice of a polybenzimidazole (PBI) film that is not so desirable. The straight thin lines running from top left to bottom right are the results of a non-smooth diamond knife edge, and the wavy curves vertical to them with larger spacings result from the intrinsic mechanical instability of the film.

### 3.2. Drop Casting, Physical Adsorption, In Situ Sample Growth, and Substrate Selection

Direct dispersion or in situ growth on ultrathin carbon or silicon substrates may be suitable for samples in their powder or solution form [8,20]. Powder samples or samples in non-solvents are typically dispersed at 1% by weight, while soluble samples are typically dispersed at 0.1–0.5% by weight since they tend to have better film coverage [6,7,8,9,10,11,13,14,17,20,22]. Dispersions in non-solvents typically undergo an ultrasonication of a few minutes so that larger aggregations can break apart before settling onto the TEM grid substrates. Direct physical adsorption is also possible by dipping TEM grids with carbon films into powder-form samples. Even a very slight visual color change on the carbon film suggests plenty of physical adsorption.

The current generation of commercially accessible TEM substrates offers significantly more options than a decade ago. This made in situ formation of samples on TEM grids possible, with or without in situ TEM. For example, chemically active functional end groups of polymers, silanes, or other organic species can thus be covalently bonded to a silicon window on TEM grids that is both electron-transparent and robust [14].

Holey carbons are great substrates on TEM grids because many parts of the sample can be hung in vacuum for minimal background interferences, while continuous ultrathin carbon films provide support for fragile and highly beam sensitive samples. Substrate films with large openings are suitable for a sample that needs continuous imaging without crossing metal grid bars but will suffer undesirable sample movements at higher magnifications.

### 3.3. Plunge Freezing

Samples involving liquid-form nanostructures require a fast freezing of the liquid before undesirable solvent crystal formation [6,9,20]. The plunge freezing, cryo-transfer of the sample onto TEM grids, and subsequent cryo-TEM experiments should be performed under continuous protection of liquid nitrogen to ensure the whole process is maintained at the liquid nitrogen temperature [6,9,20]. For aqueous samples, a hydrophilic treatment on the TEM grid may be necessary to enhance sample wetting [6,9,20]. A cryo-chamber surrounding the sample stage inside the cryo-TEM is often necessary to maintain the sample temperature stable at the liquid nitrogen temperature, in addition to a liquid nitrogen-protected cryo-sample holder.

### 3.4. To Stain or Not to Stain

As mentioned earlier, the polymer and soft complexes that are discussed here are fundamentally distinguishable when compared to proteins and other subjects of biologic cryo-TEM, in terms of polydispersity and morphological consistency [6,8,9,20]. The latter relies on the staining and monodispersity of biological macromolecules to an evenly distribute electron dose in a large number of identical crystals for enhanced contrast.

Selective staining can be useful if no other contrast mechanism is sufficient for identifying the nanostructures of interest [6,8,9,20]. For example, a composite system may have a nanostructured component with double bonds, which will promote the adsorption of heavy ruthenium or osmium staining agents [6,8,9,20]. A crystalline domain with dense packing can resist the staining agent more than its amorphous counterpart [6,8,9,20].

In other cases, it may not be desirable to stain the synthetic polymers and soft complexes, since the staining agent can swell domains and cause chemical changes [6,8,9,20]. SAED, EDX, and EFTEM results may not be accurate or possible anymore. The staining-induced morphology should be monitored as a function of the staining levels so that the pristine structures can be differentiated from the stain-caused effects.

### 3.5. Sample Drifting

Sample drifting in organic samples is a practical challenge that requires additional attention. The causes of larger sample drifting in covalently or weakly bonded materials as compared to inorganics can be multifold. These include large structural changes during electron beam exposure, charge accumulation without dynamically compensable electrical conductivity, and localized sample heating coupled with poor thermal conductivity [6,7,8,20]. Baking the sample with a lower current density at a low magnification can help stabilize the region. A robust substrate or thin protective layer will be beneficial too. Mitigation of sample drifting can be especially critical for ultrahigh resolution imaging, or experiments requiring a longer data collection time such as three window-based elemental mapping [6,7,8,9,20].

## 4. Electron Dose and Spatial Resolution

For synthetic polymers and soft complexes, a balance should be struck between the target spatial resolution and the electron dose used for imaging or diffraction. It is known that, given other conditions are constant, when the magnification increases by 10 times, the electron dose delivered will increase by 100 times [8]. The impact of electron dose-induced changes will be more and more severe as the magnification increases towards the limit of spatial resolution. The magnification-dependent total dose is described as follows:

(2) $$D_{t}={j\;M}^{2}t,$$

where *j* refers to the current density on the screen in the unit of pA/second, *M* is the magnification of the microscope, and *t* is the exposure duration in the unit of second.

According to tens of data points collected in reference [8], the thermal stability of the organic material or polymer has a logarithmic relationship with its total end-point dose or critical dose:

(3) $${\mathrm{Log}D}_{c}=\frac{T_{m}}{A}-B,\;$$

where $T_{m}$ is the melting temperature of the organic material or polymer, and A and B are two constants. The values of constants A and B are experimentally determined to be about 150 and 5.7, respectively [8].

### 4.1. Bright Field and High Resolution

An increasing acceleration voltage between 5 and 400 kV will enhance the penetration power of the electron beam, which also corresponds to a higher critical electron dose for polymers and organic materials [8]. This also means that the electron–sample interaction is stronger per single electron at 5 kV (as compared to 400 kV), yielding higher contrast per electron, causing more damage, and allowing less electrons to travel across the sample thickness. On the other hand, at higher voltages such as 400 kV, the electron–sample interaction is weaker per single electron, yielding lower contrast and causing less damage individually, but allowing more electrons to travel across the sample thickness. In other words, the contrast at a lower kilovolt value is realized by a smaller amount of electrons, where each has a larger electron–sample interaction, and the contrast at a higher kilovolt value is driven by a larger amount of electrons, where each has a smaller electron–sample interaction [8].

The following four steps highlight a routine procedure for low-dose high-resolution TEM [7,8]:

- Calibration of the survey, focus, and image mode;
- Search for areas of interest in the survey mode (low magnification, low dose, and low resolution);
- Focus on a neighborhood area near the region of potential interest in the focus mode (at a high magnification equivalent to that of the image mode);
- Go to image mode and allow the system to automatically take images using the designed exposure time;
- Thickness variation, sample drifting, and the misalignment between the survey, focus, and image mode can contribute to a failed attempt. Each new sample may require a new adjustment in imaging parameters.

### 4.2. Diffraction

The following steps are summarized for low-dose selected area electron diffraction [6,8,9,20]:

- Set a proper camera length. Polymers and organic crystals typically have much larger unit cells than those of ceramics and metals.
- Perform calibration by acquiring a diffraction pattern at the camera length of choice for a sputtered or commercially available gold (or aluminum) film. Align the beam stop so that the pattern is free from the effect of the overly bright central beam. Gold (111) rings should cover a large portion of the pattern to allow features from polymers and organic crystals to show up in detail.
- Angular relations between the SAED pattern and corresponding bright-field image should be understood before acquiring SAED patterns from unknown samples.
- Navigate sample position XY in diffraction mode after Z height adjustment and focusing.
- Observe fading of SAED patterns under the electron beam.
- Capture fading SAED patterns under the electron beam in the context of the electron beam dosage or exposure.
- Real-space imaging at the same location.

## 5. Chemical Mapping with Monitored Electron Damage

For EELS, signals of interest may include a low-eV plasmon region (for example, in organic semiconductors) or core edges of C, F, S, N, O, and some other minor elements available in synthetic polymers and organic complexes. The following procedure is proposed for minimizing electron dose damage in electron energy loss spectroscopy and energy-filtered TEM [6,8,10,20,23]:

- EELS spectrum calibration at 0 eV with minimal brightness and exposure time.
- Choose either a low-eV plasmon region or core edges.
- Acquire an elastic image for a region of interest as a baseline before EELS scans. Monitor the changes after various EELS scans.
- Spectroscopic measurements with beam damage monitoring.
- Acquire another elastic image for a region of interest as a baseline before EFTEM mapping.
- Use a minimal brightness and exposure time as a starter for EFTEM mapping. Blank the beam, or turn down the brightness right after the mapping to prevent the sample area from receiving an additional electron dosage.
- Monitor the changes in elastic images.
- Contrast improvements by gradually increasing the brightness and exposure time.
- Continued monitoring of real-space images.
- Estimate an optimal brightness and exposure time combination, in order to minimize beam-induced changes and maximize EFTEM contrast.

EELS calibration is typically performed with an exposure time of milliseconds, which may not lead to noticeable beam-induced changes. High-quality EELS scans on synthetic polymers and organic complexes, however, can cause significant morphological changes since a detail-oriented accumulative acquisition of the EELS spectrum may require a large boost in the beam brightness as well as the duration of exposure times. The optimal exposure time for EFTEM mapping on synthetic polymers and organic complexes is often in the range of seconds. A longer exposure time in EFTEM mapping may require more sample stability and drift correction after image acquisition, in addition to damage monitoring.

## 6. Other Low-Dose Techniques and Temporal Resolution

In addition to the low-dose techniques that are described above, other approaches are becoming available with automation and advanced time-resolved equipment. For example, a high-speed camera such as K3 from Gatan can offer hundreds or even thousands of frames per second [26]. Pulsed laser-based systems use pump-probe methods to study the dynamics of beam-sensitive materials in TEM, offering a stunning temporal resolution of femto- or picoseconds [27]. Their possibilities range from monitoring atomic movement during phase transitions to studying the dynamics of biological structures in environmental chambers. Alternatively, an electrical phase modulator can improve the temporal resolution of a conventional TEM to the nano- to picosecond range [28].

“Aloof” or “Leapfrog” STEM or EELS can be programmed to collect data from regions of interest on beam-sensitive samples [29,30,31]. A recent vibrational EELS study using a monochromated and aberration-corrected STEM was able to differentiate site-specific isotopic labels locally [32]. Scanning electron nanobeam diffraction [33] was used to map the orientation and crystallinity of conjugated polymers.

## 7. Morphological Variations and Hierarchical Structures

Fractal or hierarchical structures are common in synthetic polymers and soft assemblies [1,2,3,4,5,6]. In some cases, there are often more morphological variations in them than many other categories of materials. To obtain a reliable link to their functionality, one not only needs to understand their macroscopic structures but also microstructures, nanostructures, and the transitions between different length scales.

**Figure 3:**
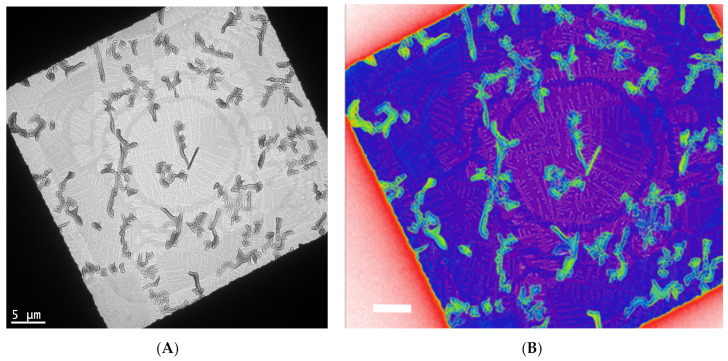
Bright-field TEM of a controlled crystallization of functional pentacene small molecules and conjugated polymers yields fractal crystal domains grown at various orientations and length scales (**A**). One layer consists of crystals with lengths of 5–10 microns, while another layer consists of crystals that are almost ten times smaller. In a color rendering based on mass thickness contrast (**B**), the group of larger crystals is in green, while the group of small crystals is around the purple region. These are unpublished results related to a previous published work [17].

In Figure 3, a controlled crystallization [17] is used to grow assemblies of functionalized pentacene small molecules and conjugated polymers. This yields fractal crystal domains grown at various orientations and length scales (Figure 3A). One layer consists of crystals with lengths of 5–10 microns, while another layer consists of crystals that are almost ten times smaller. In a color rendering based on mass thickness contrast (Figure 3B), the group of larger crystals appears in green, while the group of small crystals is around the purple region. Within the purple region, additional fractal-shaped, smaller crystals are visible.

## 8. Case Studies

Herein, selected examples of electron microscopy work from our own lab and elsewhere are grouped by the applications of synthetic polymers and soft complexes, in order to provide glimpses of recent advances and future possibilities.

### 8.1. Energy Conversion and Optoelectronics

#### 8.1.1. Polymer-Based Solar Cells

Understanding the nanomorphology is critical in order to enhance the power conversion efficiency of organic solar cells [2,5]. Film floating at the water surface, FIB, and EFTEM were used to examine the in-plane and out-of-plane nanomorphology of organic solar cell systems with low-bandgap polymers as p-type components and fullerene derivatives as n-type components [2,21,34,35,36,37]. The power efficiency of organic solar cells relies on the continuous penetration of interconnected donor- and acceptor-rich domains [38,39,40,41,42,43,44,45,46,47,48,49,50,51]. Those works are complementary to and simpler than 3D tomography [52]. Many of the low-bandgap materials contain F, N, S, O, and Si, which may be used for nanoscale EFTEM mapping.

#### 8.1.2. Organic Semiconductors

Conductive polymers and organic semiconductors including organic molecules with various chemistries, sizes, and architectures (linear, block, graft, centipedes, stars, brushes, etc.) play increasingly more important roles in sensors, photodetectors, organic light-emitting diodes (OLEDs), and organic thin-film transistors (OTFTs) [2,5,10,34,53,54,55,56,57,58,59,60,61,62,63,64,65,66,67,68,69,70,71,72].

**Figure 4:**
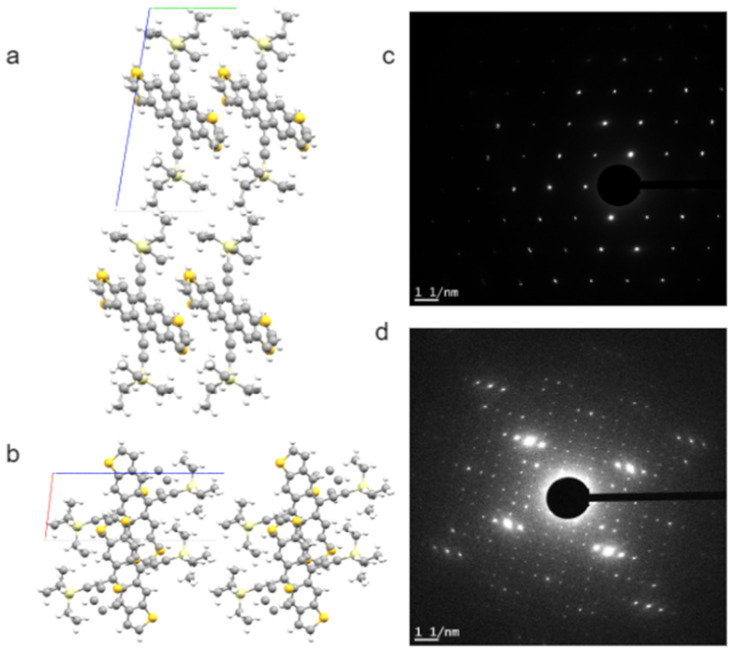
Bulk crystal structure of p-type small molecule organic semiconductor TES ADT: (**a**) viewed down a axis, and (**b**) down b axis. The a, b, and c axes of the unit cell are in red, green, and blue, respectively. This bulk crystal structure corresponds well to the [001] zone SAED patterns of TES ADT crystalline thin films grown from THF (**c**). Hexane solution will induce a polymorph that is much larger in unit cell sizes, as indicated by the [001] zone SAED patterns in (**d**). The simulated views and SAED patterns shown here are unpublished results related to the work reported in [73].

Many of these conjugated molecules are highly crystalline, which provides opportunities for SAED and HRTEM studies. The crystallinity, crystal orientation, and interconnection between crystalline and amorphous regions are often of keen interest for structure–property relationships in these energy conversion or optoelectronic devices. Figure 4 presents an example of low-dose SAED patterns from a *p*-type solution-crystallized organic semiconductor: triethylsilyl-functionalized anthradithiophene (TES ADT). A solvent-induced polymorphism of TES ADT is explained based on these SAED results [73].

#### 8.1.3. Conjugated (Co)Polymers

**Figure 5:**
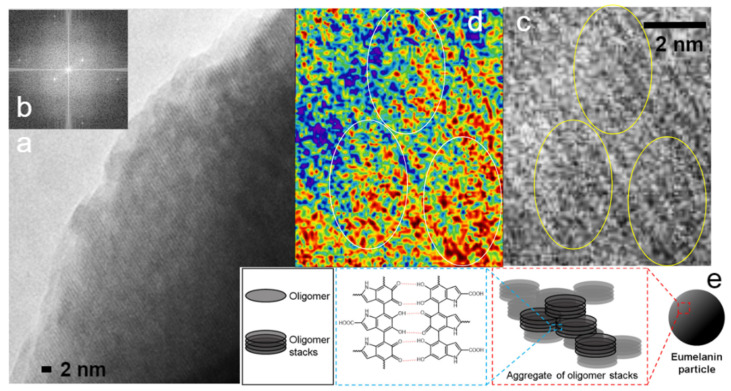
(**a**) Low-dose high-resolution TEM image of a melanin sample. The largest lattices that are visible have a spacing of 0.58 nm, which corresponds to the innermost pair of dots in the fast Fourier transformation image. (**b**) A Fourier transformation of a central region in Figure 5a. (**c**) A zoom-in of Figure 5a. (**d**) A color rendering of Figure 5c. Local crystalline packing of melanin is visible including knitted or spiral-like structures in the highlighted circles, which correspond to the fine structures inside the oligomer stack shown in Figure 5e. (**e**) The cartoon and molecular structures. Reproduced from [74] under the terms of the Creative Commons CC BY license [18]. No changes have been made.

Both synthetic and natural conjugated polymers have been of intense interest for decades thanks to their unique optoelectronic behaviors and biofunctionality [2,5,74]. Figure 5 shows a low-dose HRTEM image of a natural conjugated polymer, melanin. A possible correlation of the high-resolution TEM results and melanin’s hierarchical structure [74] is provided (Figure 5).

Block copolymers with a conjugated polymer component can be imaged without staining because of the crystal packing-induced contrast [55]. For EFTEM experiments, conjugated polymers and organic semiconductors have characteristic features at low-eV plasmon regions (e.g., 19 eV and 30 eV for *n*-type and *p*-type materials, respectively) that have much stronger EELS signals and may be used in combination with their core edges [2,21,34,35].

### 8.2. Energy Storage

#### 8.2.1. Meso/Nanoporous Materials and Membranes

Nanoscale interfaces are key to many transport-related phenomena [1,2,3,4,5,6]. For example, lattice imaging was achieved by low-dose HRTEM for porous metal–organic frameworks made from a template-free synthesis to understand their hierarchical structures and potential applications [11]. Low-dose TEM was used to examine the core–shell and pore structures of an in situ crosslinked polymer molecular sieve, as well as a mesoporous copolymer-based sorbent [75,76]. Carbon mapping of low-dose EFTEM confirmed the possibility of using liquid-like polymer matrices as a separation medium and a hollow nanostructure as a transport path for a porous liquid [77].

#### 8.2.2. Polymer Electrolyte

**Figure 6:**
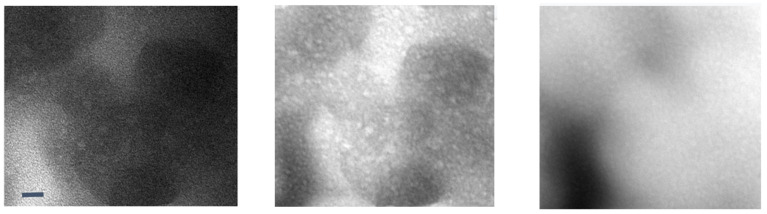
Low-loss EFTEM image series of a soluble fullerene/polyethylene oxide-based lithium-conducting solid electrolyte. From left to right: 0 eV, 30 eV, and 62 eV. The images show fullerene aggregates (white dots) with the highest contrast at an energy loss of 30 eV. These are unpublished images related to the work published in [79]. The scale bar is 10 nm.

**Figure 7:**
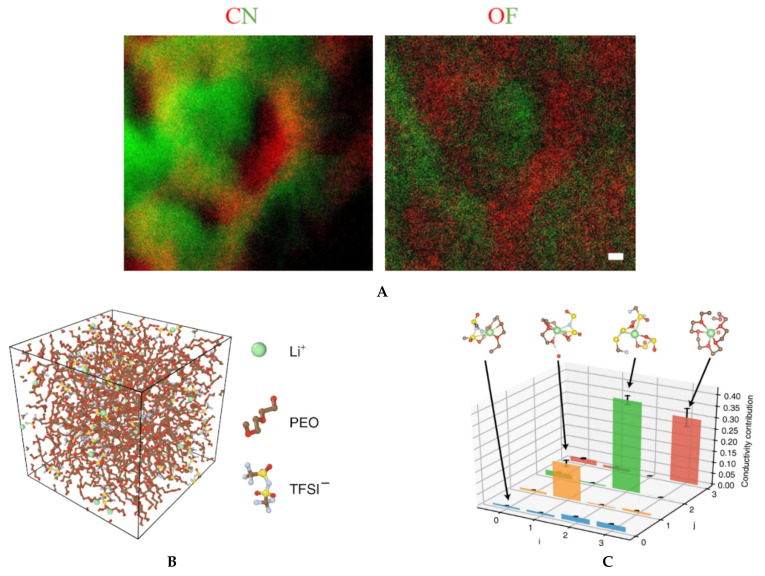
(**A**) Elemental mapping of fullerene-modified PEO/LiTFSI (10/1 by weight) solid electrolytes by low-dose EFTEM. The two colored images are: carbon map in red with nitrogen in green (left), and oxygen map in red with fluorine in green (right). The two images were taken from the same region and share the same scale bar of 20 nm. These are unpublished images related to the work published in reference [79]. (**B**) and (**C**) A four-state dynamical model learned for lithium ion in a PEO/LiTFSI polymer electrolyte. (**B**) Structure of the PEO/LiTFSI polymer electrolyte. (**C**) Contribution from each transition to lithium ion conduction. Each bar denotes the percentage that the transition from state i to state j contributes to the overall lithium ion conduction. The error bars report the 95% confidence interval from four independent trajectories in the test data. Figure 7B,C reproduced from [80], under the terms of the Creative Commons CC BY license [18]. No changes have been made.

The current generation of solid electrolytes has intrinsic limitations at the organic–inorganic interfaces as well as crystal grain–grain boundary interfaces [4,78,79]. A reciprocated cocrystallization of polyethylene oxide (PEO)/polyvinylidene difluoride (PVDF)-based solid polymer electrolytes with an enhanced grain distribution was monitored using low-dose SAED and low-dose EFTEM [78]. Another attempt to improve the grain–boundary interface was to use a fullerene nanofiller [79]. Figure 6 and Figure 7 show a series of low-dose energy-filtered TEM images from a lithium-conducting fullerene-reinforced solid polymer electrolyte. A 10% fullerene loading is found to enhance ion transport in a PEO/lithium bis (trifluoromethylsulphonyl) imide (LiTFSI) system (10/1 wt.) [79]. Figure 6 shows fullerene aggregates (white dots) with the highest contrast at an energy loss of 30 eV. Figure 7 compares the available low-dose EFTEM images of these PEO/LiTFSI composites with a new simulation result [80] that estimates a four-state dynamical model as well as contributions from each of these four transition states to lithium ion conduction in a PEO/LiTFSI electrolyte. Although the simulation shown in Figure 7 is on a much smaller length scale than that in the EFTEM images, the four transition states and their larger-scale assemblies can contribute significantly to the carbon–nitrogen or oxygen–fluoride distribution maps in Figure 7a.

#### 8.2.3. Battery Interfaces

Recent lithium battery research has benefited greatly from cryogenic STEM-related techniques. For example, the solid–liquid interphase and two types of dendrite formation in lithium metal batteries were studied by vitrifying the liquid electrolyte for chemical mapping [81]. The dendrites in carbonate electrolytes were found to grow along the <111>, <110>, or <211> directions, with different nanostructures grown in different electrolytes [82]. Electrochemically deposited lithium metal was found to be amorphous with LiF in the solid–electrolyte interface [83]. The cathode–electrolyte interface was studied with cryo-STEM and EELS to reveal the chemical nature as well as cycling and shorting effects of coating formation [26].

### 8.3. Medicine

#### 8.3.1. Macromolecular Medicine

Synthetic polymer-based systems, such as biocompatible block copolymers, responsive polymers, artificial vesicles, liposomes, polymer–drug conjugates, and protein–polymer conjugates, are making extraordinary contributions to our society [3]. Exploring their self-assembly and correlating their potential pharmaceutical performances are especially relevant when we are counting on vaccination to fight against a global pandemic.

Bulk TEM and solution-state TEM are important tools for block copolymers, combs, centipedes, stars, cyclics, peptides, and polypeptoids, which have many potential applications including in the medical field [22,84,85,86,87,88,89,90,91,92,93].

Low-dose EFTEM was used to resolve the structures of photo-crosslinkable poly(propylene fumarate) (PPF)-co-polyhedral oligomeric silsesquioxane biodegradable nanocomposites for bone repair applications [13]. Low-dose elastic images and carbon maps were able to compare the antibody immobilization on COOH, polyethylene glycol, and a dendrimer-functionalized silicon substrate, which contributes to a novel method for cancer detection [14].

#### 8.3.2. Self-Assembly and Kinetics of Block or Graft Copolymers

Recently, variable-temperature liquid cell TEM was used to monitor reversible polymerization and polymerization-induced self-assembly (PISA) [88]. This new method can provide many opportunities for studying kinetic solution assembly or monitoring polymer reactions.

With the help of electron microscopy, polyacrylic acid (PAA)-*b*-polyisoprene (PI)- and polyacrylic acid-*b*-polystyrene(PS)-based solution assemblies formed vesicle–cylinder nanoparticles, multicomponent vesicles, and cylinder–disk particles under kinetic control [91]. Multicompartmental micelles of PS-*b*-PI-*b*-PAA triblock terpolymers were formed by aqueous assembly [85]. Cryo-microtome and selective staining were used to examine the polymer architectural effects on the phase morphology of PS-*b*-PI copolymers [92]. Microphase separation was observed in comb- and centipede-type copolymers of poly(n-butyl acrylate)-*g*-PS [22]. Solution self-assembly of triblock polymers with fluorinated polyhedral oligomeric silsesquioxane demonstrated cylinders, nanosheets, and patterned vesicles in sequence with increasing water content [93].

#### 8.3.3. Self-Assembly and Kinetics of Biomacromolecules

It may not be possible to highlight all of the important progress in this category. However, a few examples are provided here. Cryo-TEM was used to study glucose-based coil–brush block copolymers as a function of the brush and coil length [89]. In addition, an onion-type dendrimersome was achieved with a controlled size and number of internal bilayers [94].

Computationally designed 29-residue peptides were found to serve as building blocks for nanostructured lattice formation [90]. Collagen-like peptides formed nanosheets via solution assembly [95]. Low-dose SAED was used to characterize coil-crystalline diblock copolypeptoids [96].

#### 8.3.4. Small Molecule Drugs

Polymorphs and crystal structures of small molecule drugs are of paramount importance to their bioavailability and pharmaceutical functions [97,98,99,100], which potentially involve a large amount of research expense or economical loss.

For pharmaceutical crystals that are too small for X-ray studies or too difficult to use for the growth of single crystals of large sizes, low-dose electron diffraction is favored. For example, 3D electron diffraction and density functional theory are used to perform structural analysis on the metastable loratadine form II [98]. Chiral isomers are associated with their bioactivities, and a dynamic refinement of low-dose electron diffraction on micrometer crystals enabled absolute structure determination [99]. In addition, 3D precession electron diffraction tomography was used to solve the crystal structure of two important pharmaceutical drugs, ramelteon and tolvaptan [100]. Low-dose high-resolution TEM was able to provide important insights on a pharmaceutical small molecule drug, furosemide, because its defects, crystal structures, and polymorphism can play key roles in the dissolution rate and formulation process [97].

## 9. AI (Artificial Intelligence) for Soft Nanostructure Analysis

**Figure 8:**
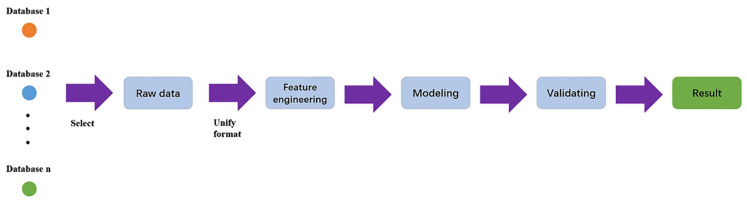
Machine learning workflow. Reproduced from [106], under the terms of the Creative Commons CC BY license [18]. No changes have been made.

**Figure 9:**
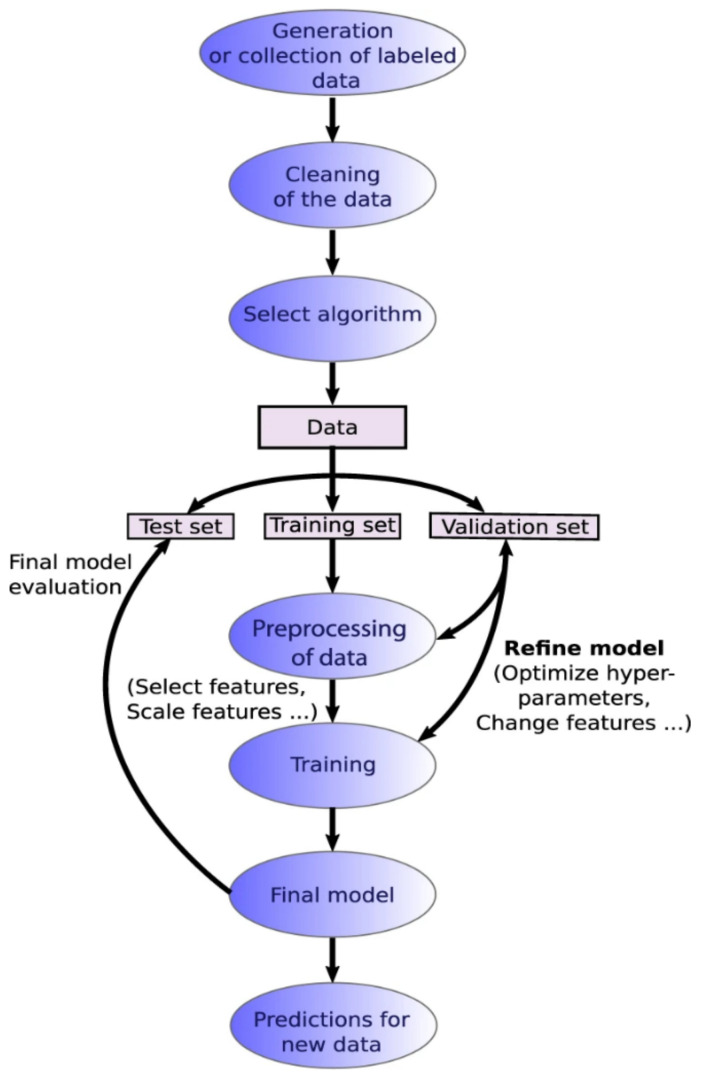
Supervised learning workflow. Reproduced from [107], under the terms of the Creative Commons CC BY license [18]. No changes have been made.

Data analysis and machine learning application packages [101,102,103,104,105] have become more readily available for various microscopy aspects including STEM, SEM, fluorescence microscopy, and holography. A typical machine learning process is outlined in Figure 8, which highlights the workflow from database formation or data collection to data cleaning, choice of algorithms for modeling, evaluation and validation of the chosen model, and, finally, applying the model to generate new insights [106]. Machine learning can be an unsupervised or supervised process, based on the involvement of data labeling or human inputs [107]. A supervised learning process is outlined in Figure 9 [107].

The nature of beam-induced sample changes in organic and polymeric materials is rooted in the chemistry and production of these materials, which largely relies on weak forces and covalent bonds to build up the ordered assembly and crystalline packing [7,8,9,19,30,108]. Due to the breakage of covalent bonds and loss of weak interactions upon overdosed electron–sample interactions, beam-induced morphological changes can take a few different routes, including carbon buildup, increased crystalline defects, shrinking of crystal dimensions, melting, crosslinking, bond breakage, mass loss, and ruptures [7,8,9,19,30,108]. Some of these processes can be easily mixed with the real features of the pristine sample. If one can properly use machine learning to train a program on how to differentiate between beam-induced effects and real features during or after experiments, the current generation of low-dose electron microscopy techniques can be greatly improved.

**Figure 10:**
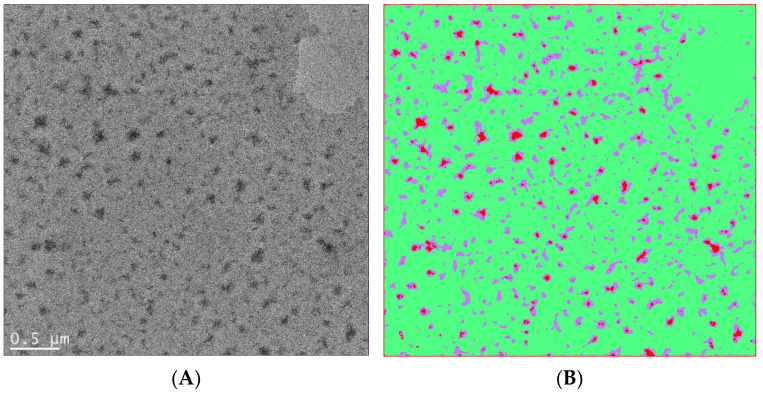
(**A**) Bright-field TEM image of a polyvinylidene fluoride (PVDF)-block-polystyrene (PS) block copolymer film showing crystalline domains in darker regions. The volume fraction of the PVDF block is 20% in the PVDF-b-PS copolymer. (The copolymer was provided by Alex Asandei, University of Connecticut.) (**B**) An attempt to classify PVDF-b-PS crystalline domains (red) and amorphous domains (green), as well as their interfaces (purple), by using the Fiji package of Image J and the machine learning capability of the Weka plugin. The Weka program is based on [102]. The Weka program was trained by user-predefined regions and then used a chosen algorithm to automatically classify each pixel. For the case of PVDF-b-PS, a fast random forest method was used to perform image segmentation, which also yields probability maps and threshold curves for performance evaluation.

**Figure 11:**
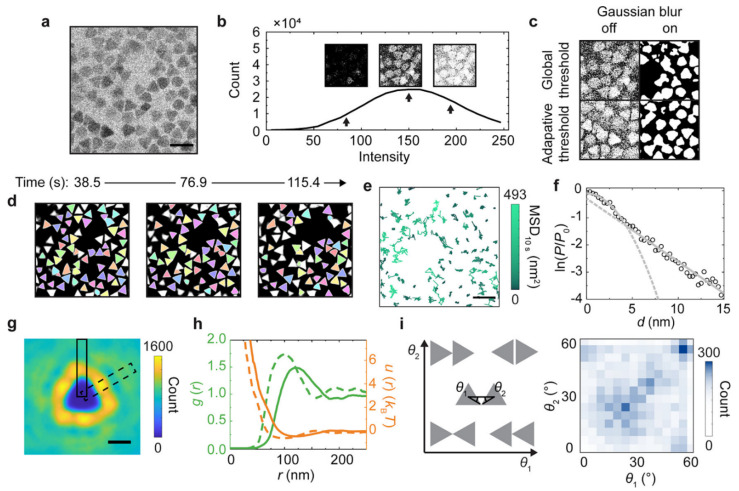
Statistical analysis of the diffusion, structure, and interaction landscape of gold triangular nanoprisms imaged by liquid-phase TEM, enabled by accurate segmentation based on the trained U-Net [109] model. (**a**) Raw liquid-phase TEM snapshot of gold triangular nanoprisms taken at a dose rate of 3.7 e^–^·Å^–2^·s^–1^ in a SiNx liquid chamber. (**b**) Histogram of the pixel intensity of the liquid-phase TEM image (**a**) which shows a single-peaked distribution (black line) without a clear valley differentiating the nanoprisms and the background. Insets: Binarized images based on different signal intensity thresholds as denoted by the arrows. (**c**) Binarized images of (**a**) different combinations of Gaussian blur and thresholding by built-in functions in MATLAB. (**d**) Tracked nanoprisms (the same color denoting the same nanoprism) in time-lapse liquid-phase TEM images based on U-Net predictions. (**e**) Temporal trajectories of the tracked centroid positions of nanoprisms colored by their mean squared displacement (MSD) at a time interval of 10 s. (**f**) Natural logarithm of the relative probability ln(P/P0) of displacements of the tracked nanoprisms (**d**) at a time interval of 0.77 s with a parabola fitting at small displacements and a linear fitting at large displacements, where P denotes the probability of a displacement of d, and P0 denotes the probability of d = 0. Dash lines denote the extended fitting results. (**g**) The 2D distribution map of the occurrence of other nanoprisms when one nanoprism is positioned at the coordinate center. (**h**) The radial distribution function and “effective” interaction landscape based on the Boltzmann inversion rule, as derived from (**g**). Dash lines in the plot correspond to data from the dashed box in (**g**), and solid lines correspond to data from the solid box in (**g**). (**i**) The 2D histogram (right) of the relative orientations (defined by θ1 and θ2 combined, left) of two nanoprisms at a particle center-to-center distance r < 200 nm. Scale bars: 200 nm in (**a**) and (**e**); 100 nm in (**g**). Reproduced from [110], under the terms of ACS AuthorChoice License.

In Figure 10, an example of using artificial intelligence to analyze soft matter TEM results is shown, which involves user-defined segmentation as training sets and automated segmentation with an algorithm of choice (such as the fast random forest model used in this case). In another example, a convolutional neutral network algorithm, U-Net [109], was combined with liquid-phase TEM to study the dynamics of surfactant-treated nanoparticles, which require careful monitoring of the accumulated electron doses [110] (Figure 11). This work showcased the possibility of using machine learning in soft matter TEM for automated physical insight generation, which goes far beyond feature recognition and image segmentation.

In addition, machine learning may be coupled with low-dose electron diffraction for crystal symmetry determination [111], and its application in beam-sensitive materials can have a large impact on organic crystals such as organic semiconductors, organic battery electrodes, and pharmaceutical crystals.

## 10. Outlook

**Figure 12:**
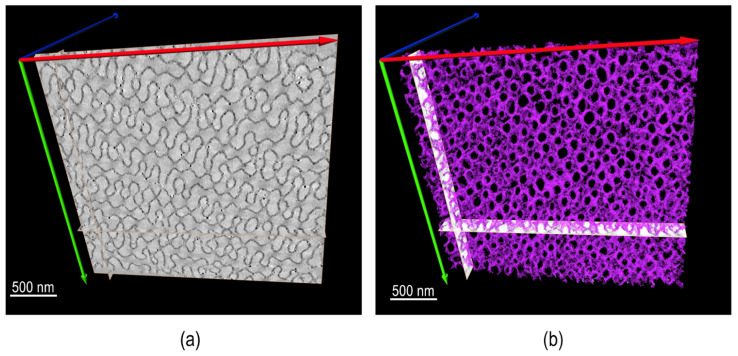
PS-b-PAA porous particles: (**a**) TEM image and (**b**) TEM tomography reconstruction of a 100 nm slice. Reproducedfrom [113], under the terms of the Creative Commons CC BY license [18]. No changes have been made.

**Figure 13:**
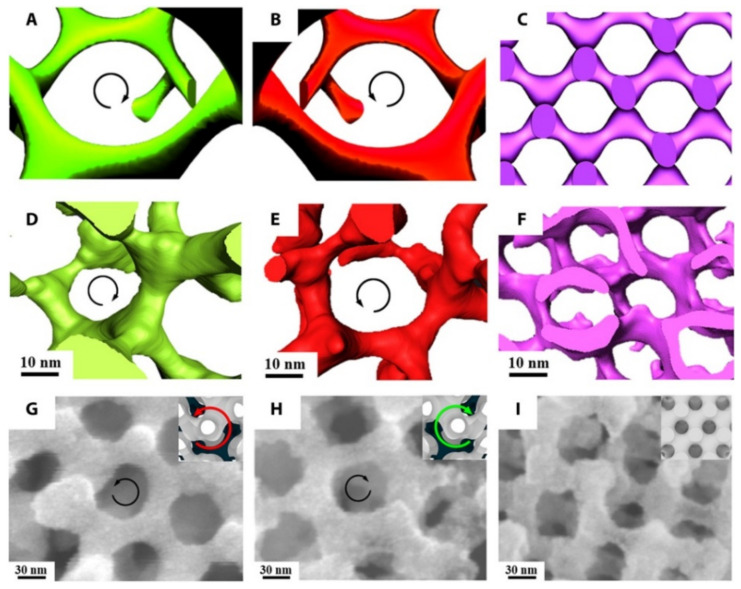
3D imaging of a PI network and SEM images of a Ni network fabricated from templating a polylactide network. (**A**–**C**) Simulated images and (**D**–**F**) 3D reconstructions from electron tomography of PI-b-PS-b-PLLA, PI-b-PS-b-PDLA, and PI-b-PS-b-PLA, respectively. Direct visualization of the right-handed [112] helical locus of the PI network in (**A**) and (**D**) and the left-handed helical locus of the PI gyroid network in (**B**) and (**E**). When an achiral PLA block is used, the structure is an achiral diamond (**F**). (**G**–**I**) FESEM images of the Ni network from PI-b-PS-b-PLLA, PI-b-PS-b-PDLA, and PI-b-PS-b-PLA, respectively, at which the Ni network takes on the handedness and volume fraction of the particular polylactide block that is hydrolyzed. Insets show the corresponding simulated structures with dark core struts as a visual guide. Direct visualization of the Ni gyroid network along the [111] direction demonstrates a left-handed locus in PI-b-PS-b-PLLA (**G**) and right-handed helical locus in PI-b-PS-b-PDLA (**H**). Reproduced from [114], under the terms of the Creative Commons CC BY license [18]. No changes have been made.

An additional important direction of using TEM for synthetic polymers and soft complexes lies in tomography. A recent review paper covered this topic extensively [112]. Figure 12 is an example of using electron tomography to study the 3D porous structure of polystryrene-*block*-polyacrylic acid (PS-*b*-PAA) particles [113]. More examples of using tomography and modeling to resolve polymer nanostructures are provided in Figure 13 [114].

**Figure 14:**
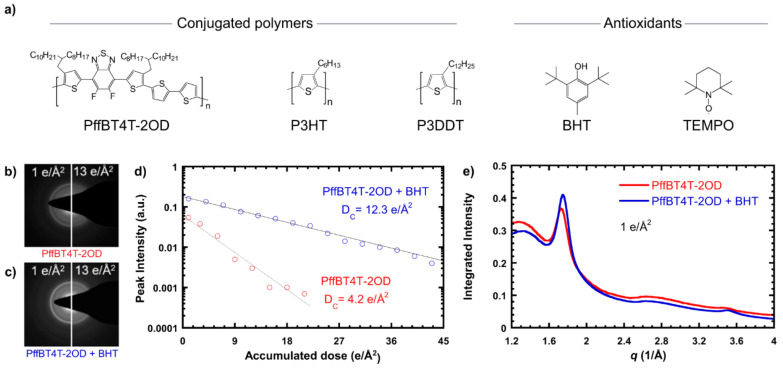
(**a**) Chemical structures of conjugated polymers and antioxidants used in the study of [115]. (**b**) Electron diffraction pattern of neat PffBT4T-2OD at low and high doses, showing loss of crystal structure after an accumulated dose of 13 e/Å^2^. (**c**) Electron diffraction pattern of PffBT4T-2OD + BHT at low and high doses, showing a partially preserved crystal structure at 13 e/Å^2^. (**d**) Background-subtracted peak intensity vs. accumulated dose for PffBT4T-2OD and PffBT4T-2OD + BHT, with exponential fits showing the persistence of the crystal structure at a higher dose with the addition of BHT. (**e**) Azimuthal integration of electron diffraction patterns of PffBT4T-2OD with and without BHT at a dose of 1 e/Å^2^, showing no change in the crystal structure. Reproduced from [115], under the terms of the Creative Commons CC BY license [18]. No changes have been made.

**Figure 15:**
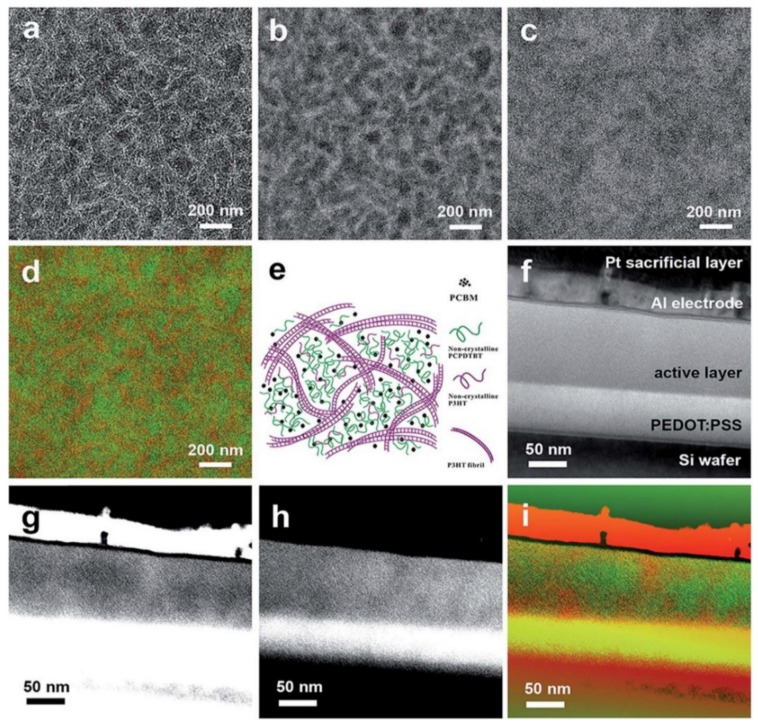
EF-TEM (**a**) 0 ± 4 eV, (**b**) 19 ± 4 eV, and (**c**) 30 ± 4 eV images, (**d**) color mix map for lateral section of pre-annealed ternary blend thin films of P3HT-32k:PCPDTBT:PCBM with a weight ratio of 3:1:4 (25 wt% of PCPDTBT), and the corresponding scheme (**e**). EF-TEM [49] (**f**) 0 ± 4 eV, (**g**) 19 ± 4 eV, and (**h**) 30 ± 4 eV images, and (**i**) color mix map for cross-section of post-annealed ternary blend thin films of P3HT-32k:PCPDTBT:PCBM with a weight ratio of 3:1:4. Reproduced from [49] with permission from the Royal Society of Chemistry.

Organic small molecules and polymers form intriguing assemblies and complexes [1,2,3,4,5]. In an example shown in Figure 14, the addition of organic small molecules in polymers serves the purpose of enhancing polymer characterization [115]. In another case, a ternary soft complex facilitates the formation of promising novel nanostructures for performance enhancement (Figure 15) [49]. In either case, low-dose TEM remains a powerful and indispensable means to probe these soft complex structures and guide their applications in improved materials processes.

In conclusion, electron microscopy continues to serve the goals of advancing synthetic polymers and soft complexes by providing nanomorphological insights at interfaces and surfaces, in bulk and in solution. These insights provide intimate connections to their potential functionality. For synthetic polymers and soft complexes, there are four main characteristics that differentiate them from their inorganic or biomacromolecular counterparts in electron microscopy studies: (1) lower contrast, (2) abundance of light elements, (3) polydispersity or nanomorphological variations, and (4) large changes induced by electron beams. This article updates the recent progress in using soft matter TEM to advance our understanding of synthetic polymers and soft complexes. Artificial intelligence is expected to reinvigorate the field by bringing in improvements in soft matter TEM analysis.
